# Comparative analysis of plant immune receptor architectures uncovers host proteins likely targeted by pathogens

**DOI:** 10.1186/s12915-016-0228-7

**Published:** 2016-02-19

**Authors:** Panagiotis F. Sarris, Volkan Cevik, Gulay Dagdas, Jonathan D. G. Jones, Ksenia V. Krasileva

**Affiliations:** The Sainsbury Laboratory, Norwich Research Park, Norwich, UK; The Genome Analysis Centre, Norwich Research Park, Norwich, UK; Division of Plant and Microbial Sciences, School of Biosciences, University of Exeter, Exeter, UK

**Keywords:** Genomics, Plant innate immunity, NLRs, Integrated domains, Gene fusions

## Abstract

**Background:**

Plants deploy immune receptors to detect pathogen-derived molecules and initiate defense responses. Intracellular plant immune receptors called nucleotide-binding leucine-rich repeat (NLR) proteins contain a central nucleotide-binding (NB) domain followed by a series of leucine-rich repeats (LRRs), and are key initiators of plant defense responses. However, recent studies demonstrated that NLRs with non-canonical domain architectures play an important role in plant immunity. These composite immune receptors are thought to arise from fusions between NLRs and additional domains that serve as “baits” for the pathogen-derived effector proteins, thus enabling pathogen recognition. Several names have been proposed to describe these proteins, including “integrated decoys” and “integrated sensors”. We adopt and argue for “integrated domains” or NLR-IDs, which describes the product of the fusion without assigning a universal mode of action.

**Results:**

We have scanned available plant genome sequences for the full spectrum of NLR-IDs to evaluate the diversity of integrations of potential sensor/decoy domains across flowering plants, including 19 crop species. We manually curated wheat and brassicas and experimentally validated a subset of NLR-IDs in wild and cultivated wheat varieties. We have examined NLR fusions that occur in multiple plant families and identified that some domains show re-occurring integration across lineages. Domains fused to NLRs overlap with previously identified pathogen targets confirming that they act as baits for the pathogen. While some of the integrated domains have been previously implicated in disease resistance, others provide new targets for engineering durable resistance to plant pathogens.

**Conclusions:**

We have built a robust reproducible pipeline for detecting variable domain architectures in plant immune receptors across species. We hypothesize that NLR-IDs that we revealed provide clues to the host proteins targeted by pathogens, and that this information can be deployed to discover new sources of disease resistance.

**Electronic supplementary material:**

The online version of this article (doi:10.1186/s12915-016-0228-7) contains supplementary material, which is available to authorized users.

## Background

Plants recognize pathogens through an innate immune system that monitors pathogen-associated molecules either outside or inside the plant cell [1–4]. Pathogen-derived molecules known to trigger immunity are commonly classified into pathogen-associated molecular patterns (PAMPs), such as bacterial flagellin or fungal chitin, which are usually presented in the apoplastic space, and pathogen-derived effectors, which are more diverse and often translocated inside the host. Effectors are commonly deployed by the pathogen to target intracellular host proteins for effective nutrient delivery or suppression of plant defense responses. The two major branches of plant immunity, PAMP-triggered immunity (PTI) and effector-triggered immunity (ETI), are defined based on the type and location of the receptor, the molecule(s) detected, and downstream signaling components. PTI commonly employs receptor-like kinases or receptor-like proteins that detect PAMPs outside of plant cells and transmit signals within the cell via phosphorylation cascades that involve mitogen-activated protein kinase signaling cascades and other protein kinases [5, 6]. ETI is initiated by plant receptors called nucleotide-binding leucine-rich repeat (NLR) proteins, which detect the presence of pathogen-derived effectors within plant cells and activate defense via as yet poorly understood mechanisms [2, 4]. Since one of the functions of the effectors inside plant cells is to disarm plant defense responses, there is a constant evolutionary arms race between pathogen effectors and components of plant immunity. This puts immense selection on pathogen effector genes [7–9] and on the effector targets and immune receptors in the plant [10–12]. Plant receptors evolve rapidly via various mechanisms, including point mutations, gene duplications and gene rearrangements [13, 14].

NLR-encoding genes are found from flowering plants to mosses [15–17]. All NLRs share a central nucleotide-binding (NB) domain, corresponding to the NB-ARC domain in Pfam. The NB domain is usually, but not always, associated with carboxy-terminal leucine-rich repeats (LRRs) and amino-terminal coiled coil (CC) or Toll/interleukin-1 receptor/resistance protein (TIR) domains [13, 18]. Although NLRs derive their name from having both NB and LRR domains, there have been several reports of disease resistance genes encoding proteins that lack LRRs [16, 19, 20]. Moreover, analyses of *Arabidopsis thaliana* RRS1 and rice (*Oryza sativa*) RGA4/Pik-1 have revealed the functional significance of additional domains present in some NLR proteins [21–25]. Therefore, plant NLRs support flexible architectures, perhaps to enable recognition of a broader range of pathogen-derived molecules.

Effectors can be recognized either through direct interaction with the NLR receptor (direct recognition) or through monitoring of an effector’s activity on host proteins (indirect recognition) [4]. Although originally sparse, reports of the direct interaction between NLR and effector proteins have been growing in recent years, and include NLR proteins encoded by the rice *Pi-ta*, *RGA5* and *PiK* genes [24–26, 27], the *Nicotiana tabacum N* gene [28], the flax *(Linum usitatissimum) L5*/*L6* and M genes [29, 30], the *Arabidopsis RPP1* gene [31], and potato (*Solanum tuberosum*) *Rpi-blb1* [32]. Indirect recognition has been well-demonstrated for many immune receptors [33–36]. In this case, the receptor protein monitors host proteins, known as “guardees” if they actively contribute to immunity or “decoys” if they mimic the authentic host target. Binding and/or modification of such a guardee/decoy by an effector leads to activation of the NLR receptor [37]. For example, the status of RIN4 protein (RPM1 interacting protein 4) is monitored by at least two independent Arabidopsis NLRs, RPS2 and RPM1, which detect cleavage or phosphorylation of RIN4 by bacterial effectors AvrRpt2 and AvrRpm1 (or AvrB), respectively [34, 38, 39]. Similarly, an Arabidopsis NLR protein RPS5 detects cleavage of a protein kinase PBS1 by bacterial cysteine protease effector AvrPphB [40]. A tomato (*Solanum lycopersicum*) protein kinase Pto interacts with effector AvrPto and is guarded by NLR protein Prf [41, 42].

Recent findings show that an NLR and a host protein involved in indirect recognition can be fused together. Specifically, NLR receptors can carry an additional protein domain, enabling perception of pathogen effectors. Such recognition mode is known as “the integrated decoy/sensor” model [43, 44] and is based on three examples of NLRs with integrated domains (NLR-IDs) and mechanistic insights into their activity: Arabidopsis NLR protein RRS1 carries an additional WRKY domain [21, 22]; and rice RGA5 and Pik-1 proteins are fused to heavy metal-associated (HMA, also known as RATX1) domains [23–25]. The acetyltransferase effector PopP2, from the wilt pathogen *Ralstonia solanacearum*, and the effector AvrRps4, from the leaf pathogen *Pseudomonas syringae* pv. *pisi*, are both recognized upon their interaction with or modification of the WRKY DNA-binding domain of RRS1 protein. Furthermore, both effectors target several WRKY transcription factors in Arabidopsis, which indicates that the RRS1-WRKY domain has evolved as a trap for the perception of effectors that target WRKY transcription factors. Similarly to RPS4/RRS1, the rice CC-NB-LRR receptor pair RGA4/RGA5 recognizes two unrelated effectors, AVR-Pia and AVR1-CO39 of *Magnaporthe oryzae*, upon their direct interaction with the C-terminus of RGA5 [27]. Interestingly, the recognition of both effectors by RGA5 occurs through a small C-terminal HMA domain, also related to the cytoplasmic copper chaperone RATX1 from *Saccharomyces cerevisiae* [27]. As for RGA4/RGA5, the CC-NB-LRR receptor pair Pik-1/Pik-2, which contains the HMA domain fused between the CC and the NB-ARC regions of Pik-1, binds Avr-Pik effector of *M. oryzae* to activate immunity [23–25]. However, to date there are no published reports of other HMA domain proteins being targeted by AVR-Pia, AVR1-CO39 and AVR-Pik, although rice Pi21 is a HMA protein that confers susceptibility to the rice blast fungus [45].

The availability of sequenced plant genomes allowed us to test if integration of new domains in NLRs is widespread in angiosperms. We have examined NLR domain architectures from 40 publicly available plant predicted proteomes, and identified 720 NLR-IDs that involved both recently formed and conserved or recurrent fusions. A previous screen performed by Cesari et al. revealed a total of 22 unique integrated-domain fusions to NLR proteins [43]. This was based on a BLAST search carried out using two previously identified NLR proteins, RGA5 and RRS1, as “baits”. This work formed an important preliminary basis for the current study. Here, we have built a high-throughput reproducible pipeline that can be applied to any newly sequenced set of predicted proteins for genome-wide identification of NLR-IDs. We have applied our pipeline in combination with the manual verification to 40 plant genomes, including mosses and flowering plants (monocots and dicots), to discover 265 unique NLR integrated-domains, including the ones that have been already described by Cesari et al. [43]. This is necessarily an underestimate since protein annotations of public datasets are often incomplete [46]; therefore, our easily adopted reproducible methodology is key to expanding these analyses even further once more data becomes available. We examined which NLR-IDs occurred in multiple plant families suggesting their conservation and functional significance. Availability of published effector interactome screens [47, 48] allowed us to overlay our analyses with predicted effector targets. Our analysis revealed that extraneous domains have repeatedly integrated into NLR proteins across all plant lineages. Some of the integrated domains are already known to be implicated in pathogen defense; for example, RIN4, NPR1. Other integrated domains originated from host proteins that may function in pathogen interactions, and are prime candidates for functional analysis to engineer disease-resistant plants.

## Results and discussion

### Identification of NLR proteins in plants based on the conserved NB-ARC domain

**Fig. 1:**
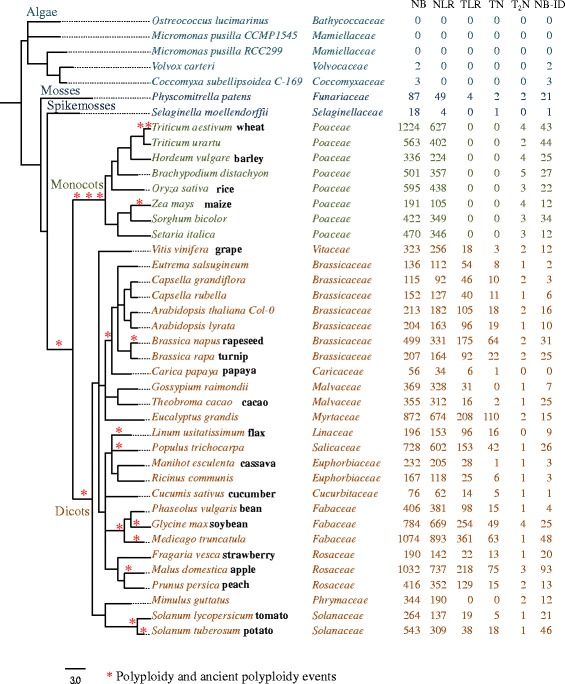
Phylogeny of the plant species and corresponding number of detected NLR and NLR-fusion proteins. The phylogeny of the plants used in the analyses was constructed using their corresponding NCBI taxon identification numbers. For the summary of NLR-IDs detected in each species, see Table 1 and Additional files 2 and 3. Annotation of all domains in NB-ARC-containing proteins and NLR-IDs and corresponding FASTA sequences are included in Additional files 4, 5, 6 and 7. NB: NB-ARC domain-containing proteins; NB-ID: NB-ARC plus any other canonical domains together with non-canonical domains; NLR: subset of NB with clearly identified LRRs; TLR: TIR-NB-ARC-LRR proteins; TN: TIR-NB-ARC proteins; T_2_N: TIR2-NB-ARC proteins

To gain insight into the evolution and diversity of NLR protein architectures across plants, we performed annotation of the Pfam NB-ARC domain-containing proteins in predicted proteomes of 40 publicly available plant species, which include algae, mosses as well as diverse families across angiosperms. (Fig. 1, Additional file 1). We have assembled a pipeline to annotate the domains present in the predicted proteomes of each species, and extracted NB-ARC-containing proteins as well as any other domain associated with it (Additional files 2 and 3). The current Pfam NB-ARC domain model (PF00931) works well for detecting NLR genes in monocots as well as dicots as it includes 151 monocot and 242 dicot species used to build the hidden Markov model. Benchmarking on Arabidopsis showed that the NB-ARC domain is specific to NLR proteins with 169 proteins detected (215 splice variants), including 149 previously published NLR sequences [13] and 20 NB-ARC-containing proteins with no LRRs, and no false positive other ATPases detected. This showed the NB-ARC domain alone is a good predictor of NLRs. The performance of Pfam NB-ARC on monocot genomes has been validated previously, i.e. Steuernagel et al. looked at sensitivity of HMMER NB-ARC searches in *Brachypodium* [49]. We filtered for the top Pfam hit for each non-overlapping protein region to ensure that only genes for which the NB-ARC domain scored higher than other ATPase-related domains were retained. As annotations of many plant species are currently fragmented, we did not require LRR presence to be a strict criterion and included all NB-containing proteins for further analyses. Altogether, we have identified 14,363 NB-ARC-containing proteins across all species (Fig. 1, Additional files 4 and 5). Of these, 720 proteins had additional domains not typical for NLR proteins (Fig. 1, Additional files 3, 6 and 7).

We have manually analyzed NLR-IDs in *Brassica napus*, *Brassica rapa*, *S. lycopersicum*, *Medicago truncatula*, *Brachypodium distachyon* and *Triticum urartu* by cross-checking the sequences against UniProtKB and Swiss-Prot databases, and were able to validate the accuracy of >95 % of high-throughput predictions (Additional file 8). Our manual analyses of NLR-IDs in wild wheatgrass (*T. urartu*) showed that there were only 3 out of 44 proteins that we predicted as NLRs and do not appear to carry a canonical NB-ARC domain showing a very low rate of false positive predictions even in genomes of monocots.

Similar to previous reports, our data show that the NB-ARC domain appears as early as mosses and is present across all surveyed angiosperms (Fig. 1). In many lineages, the increase in NB-ARC domain-containing proteins is associated with polyploidy or ancient polyploidization events (Fig. 1) [50, 51]; i.e. 1,224 NB-ARC genes in hexaploid wheat (*Triticum aestivum*), and 1,032 and 1,074 NB-ARC genes in recently duplicated apple (*Malus domestica*) and *M. truncatula* genomes, respectively [52–54]. The increase in R-genes in grasses is also likely linked to three ancient polyploidization events in its evolutionary history [50, 51]. A notable exception is maize (*Zea mays*), which contains only 191 NB-ARC proteins despite recent whole genome duplications. An unusually low number of NB-ARC-containing genes was detected in papaya (*Carica papaya*, 56 NB-ARC genes) and cucumber (*Cucumis sativus*, 76 NB-ARC genes) for which there is no clear explanation.

### Distinct class of TIR domain is present in all flowering plants

Our bioinformatics pipeline discovers any combinations of protein family domains within Pfam present together with NB-ARC. The canonical TIR-NB domain combination is present widely in association with NB-ARC in mosses as well as dicots (Fig. 1). In monocots, our analyses confirmed the absence of canonical TIR, but we discovered that a distinct related domain (Pfam domain TIR_2) is present in both monocots and dicots, and the number of family members in each species is restricted to 2–5 genes (Fig. 1). These monocot and dicot TIR2 sequences form an ancient gene family that is evolutionarily distinct from the classic TIR sequences in dicots, consistent with previous analyses suggested by Nandety et al. [20]. We propose that this family shall be recognized separately as TIR2 NLRs and not grouped with canonical TIR proteins.

It is noteworthy that TIR2 domain proteins are also present in bacteria [55] and have been studied as important virulence factors in mammalian bacterial pathogens. TIR2 domain proteins from several mammalian pathogenic species suppress animal TLR-dependent host defenses by targeting TIR2-type mammalian innate immunity proteins [56]. We have looked for and identified TIR2 domain proteins in many plant pathogenic bacteria (Additional file 9). Till now, there is no evidence regarding the role of these proteins in pathogenicity, yet the presence of TIR2 proteins both in plants and in phytopathogenic bacteria could indicate their involvement in pathogenicity similar to mammalian systems.

### Fusion of NLRs to new domains is widespread across flowering plants

We found evidence of NLR-ID fusions in mosses and across all lineages of flowering plants. The number of NLR-IDs ranged from just 1 gene in cucumber (*C. sativus*) to 93 in apple (*M. domestica*) (Fig. 1, Table 1, Additional files 2, 3, 6 and 7). The only plant with no NLR-IDs was papaya (*C. papaya*), which has a low number of 58 NLRs in total. Despite variability in the total number of NLRs across flowering plants, on average in each species NLR-IDs represented about 10 % of all NLRs and correlated with increases and decreases in total NLR numbers among species. There is a substantial variation in the number of NLRs and their integrated domains across flowering plants. However, it is hard to conclude whether there are significant differences in fusion rates across different lineages as our analyses are based on current proteome predictions for each species that may have missed or miss-annotated genes.

**Table 1:** Most prevalent integrated domains in flowering plants

Integrated domain^a^	Species	Families	Domain description
Pkinase	*A. thaliana*, *B. distachyon*, *B. napus*, *B. rapa*, *F. vesca*, *H. vulgare*, *M. domestica*, *M. guttatus*, *M. truncatula*, *O. sativa*, *P. patens*, *S. bicolor*, *S. italica*, *T. cacao*, *T. aestivum*, *T. urartu*, *V. vinifera*, *Z. mays*	*Brassicaceae*, *Fabaceae*, *Funariaceae*, *Malvaceae*, *Phrymaceae*, *Poaceae*, *Rosaceae*, *Vitaceae*	Protein kinase domain
DUF3542	*L. usitatissimum*, *M. domestica*, *M. esculenta*, *M. guttatus*, *O. sativa*, *P. persica*, *P. trichocarpa*, *S. italica*, *S. lycopersicum*, *S. tuberosum*, *V. vinifera*	*Euphorbiaceae*, *Linaceae*, *Phrymaceae*, *Poaceae*, *Rosaceae*, *Salicaceae*, *Solanaceae*, *Vitaceae*	Protein of unknown function (DUF3542)
Pkinase_Tyr	*B. distachyon*, *B. napus*, *B. rapa*, *F. vesca*, *G. max*, *H. vulgare*, *M. domestica*, *O. sativa*, *P. patens*, *S. bicolor*, *T. cacao*, *T. aestivum*, *T. urartu*, *V. vinifera*	*Brassicaceae*, *Fabaceae*, *Funariaceae*, *Malvaceae*, *Poaceae*, *Rosaceae*, *Vitaceae*	Protein tyrosine kinase
WRKY	*A. lyrata*, *A. thaliana*, *B. distachyon*, *C. grandiflora*, *C. rubella*, *G. max*, *H. vulgare*, *M. domestica*, *S. bicolor*, *S. italica*, *T. cacao*, *T. aestivum*, *T. urartu*	*Brassicaceae*, *Fabaceae*, *Malvaceae*, *Poaceae*, *Rosaceae*	WRKY DNA-binding domain
RVT_3	*F. vesca*, *G. max*, *M. domestica*, *M. esculenta*, *P. vulgaris*, *T. cacao*, *T. urartu*	*Euphorbiaceae*, *Fabaceae*, *Malvaceae*, *Poaceae*, *Rosaceae*	Reverse transcriptase-like
WD40	*B. rapa*, *M. domestica*, *M. truncatula*, *O. sativa*, *S. bicolor*, *T. cacao*	*Brassicaceae*, *Fabaceae*, *Malvaceae*, *Poaceae*, *Rosaceae*	WD domain, G-beta repeat
zf-BED	*B. distachyon*, *E. grandis*, *G. max*, *H. vulgare*, *M. truncatula*, *O. sativa*, *P. trichocarpa*, *P. vulgaris*, *S. italica*, *T. aestivum*, *T. urartu*	*Fabaceae*, *Myrtaceae*, *Poaceae*, *Salicaceae*	BED zinc finger
B3	*B. napus*, *B. rapa*, *F. vesca*, *H. vulgare*, *M. domestica*, *O. sativa*, *T. cacao*, *T. urartu*	*Brassicaceae*, *Malvaceae*, *Poaceae*, *Rosaceae*	B3 DNA-binding domain
NAM	*B. napus*, *M. domestica*, *P. trichocarpa*, *S. bicolor*, *S. italica*	*Brassicaceae*, *Poaceae*, *Rosaceae*, *Salicaceae*	No apical meristem (NAM) protein
DUF761	*C. rubella*, *C. sativus*, *L. usitatissimum*, *O. sativa*	*Brassicaceae*, *Cucurbitaceae*, *Linaceae*, *Poaceae*	Cotton fiber-expressed protein
UBN2	*M. domestica*, *T. cacao*, *T. urartu*, *V. vinifera*	*Malvaceae*, *Poaceae*, *Rosaceae*, *Vitaceae*	Gag-polypeptide of LTR copia-type
HMA	*B. napus*, *C. rubella*, *M. domestica*, *M. truncatula*, *T. urartu*	*Brassicaceae*, *Fabaceae*, *Rosaceae*, *Poaceae*	Heavy metal-associated domain
Thioredoxin	*B. distachyon*, *G. raimondii*, *H. vulgare*, *O. sativa*, *S. bicolor*, *S. italica*, *T. aestivum*, *T. urartu*, *V. vinifera*	*Malvaceae*, *Poaceae*, *Vitaceae*	Thioredoxin
VQ	*B. napus*, *B. rapa*, *C. grandiflora*, *C. rubella*, *E. salsugineum*, *F. vesca*, *M. domestica*, *O. sativa*, *T. aestivum*	*Brassicaceae*, *Poaceae*, *Rosaceae*	VQ motif
LIM	*A. thaliana*, *B. napus*, *M. domestica*, *M. truncatula*, *P. persica*	*Brassicaceae*, *Fabaceae*, *Rosaceae*	LIM domain
zf-RVT	*G. max*, *G. raimondii*, *O. sativa*, *P. vulgaris*, *T. urartu*	*Fabaceae*, *Malvaceae*, *Poaceae*	Zinc-binding in reverse transcriptase
C1_2	*B. rapa*, *O. sativa*, *T. cacao*	*Brassicaceae*, *Malvaceae*, *Poaceae*	C1 domain
DUF4219	*G. max*, *M. domestica*, *V. vinifera*	*Fabaceae*, *Rosaceae*, *Vitaceae*	Domain of unknown function (DUF4219)
EF_hand_5	*M. domestica*, *P. trichocarpa*, *T. urartu*	*Poaceae*, *Rosaceae*, *Salicaceae*	EF-hand domain pair
Myb_DNA-binding	*B. distachyon*, *E. grandis*, *R. communis*	*Euphorbiaceae*, *Myrtaceae*, *Poaceae*	Myb-like DNA-binding domain
Peptidase_C48	*F. vesca*, *G. max*, *Z. mays*	*Fabaceae*, *Poaceae*, *Rosaceae*	Ulp1 protease family, C-terminal catalytic domain
gag_pre-integrs	*M. domestica*, *T. urartu*, *V. vinifera*	*Poaceae*, *Rosaceae*, *Vitaceae*	GAG-pre-integrase domain
rve	*T. cacao*, *T. urartu*, *V. vinifera*	*Malvaceae*, *Poaceae*, *Vitaceae*	Integrase core domain
Jacalin	*B. distachyon*, *E. grandis*, *H. vulgare*, *O. sativa*, *S. bicolor*, *S. italica*, *T. aestivum*	*Myrtaceae*, *Poaceae*	Jacalin-like lectin domain
DUF3633	*A. thaliana*, *B. napus*, *M. domestica*, *P. persica*	*Brassicaceae*, *Rosaceae*	Protein of unknown function (DUF3633)
FNIP	*H. vulgare*, *M. truncatula*, *S. bicolor*, *S. italica*	*Fabaceae*, *Poaceae*	FNIP repeat
Kelch_1	*B. napus*, *H. vulgare*, *T. aestivum*, *T. urartu*	*Brassicaceae*, *Poaceae*	Kelch motif
PP2C	*F. vesca*, *H. vulgare*, *T. aestivum*, *T. urartu*	*Poaceae*, *Rosaceae*	Protein phosphatase 2C
AvrRpt-cleavage	*H. vulgare*, *M. domestica*, *O. sativa*	*Poaceae*, *Rosaceae*	Cleavage site for pathogenic type III effector avirulence factor Avr
CBFB_NFYA	*B. napus*, *B. rapa*, *L. usitatissimum*	*Brassicaceae*, *Linaceae*	CCAAT-binding transcription factor (CBF-B/NF-YA) subunit B
DUF4283	*F. vesca*, *M. domestica*, *M. truncatula*	*Fabaceae*, *Rosaceae*	Domain of unknown function (DUF4283)
F-box	*M. domestica*, *S. lycopersicum*, *S. tuberosum*	*Rosaceae*, *Solanaceae*	F-box domain
Glutaredoxin	*H. vulgare*, *S. bicolor*, *S. tuberosum*	*Poaceae*, *Solanaceae*	Glutaredoxin
PP2	*S. bicolor*, *T. cacao*, *Z. mays*	*Malvaceae*, *Poaceae*	Phloem protein 2
PPR_2	*B. napus*, *F. vesca*, *M. domestica*	*Brassicaceae*, *Rosaceae*	PPR repeat family
PRK	*G. raimondii*, *P. persica*, *T. cacao*	*Malvaceae*, *Rosaceae*	Phosphoribulokinase/uridine kinase family
U-box	*B. napus*, *B. rapa*, *F. vesca*	*Brassicaceae*, *Rosaceae*	U-box domain
UBN2_3	*M. domestica*, *T. urartu*, *Z. mays*	*Poaceae*, *Rosaceae*	Gag-polypeptide of LTR copia-type
Abhydrolase_6	*M. domestica*, *Z. mays*	*Poaceae*, *Rosaceae*	Alpha/beta hydrolase family
B_lectin	*G. max*, *V. vinifera*	*Fabaceae*, *Vitaceae*	D-mannose binding lectin
C1_3	*B. rapa*, *T. cacao*	*Brassicaceae*, *Malvaceae*	C1-like domain
Cyclin_C	*E. grandis*, *M. truncatula*	*Fabaceae*, *Myrtaceae*	Cyclin, C-terminal domain
Cyclin_N	*E. grandis*, *M. truncatula*	*Fabaceae*, *Myrtaceae*	Cyclin, N-terminal domain
DUF247	*M. domestica*, *Z. mays*	*Poaceae*, *Rosaceae*	Plant protein of unknown function
FBD	*B. napus*, *M. domestica*	*Brassicaceae*, *Rosaceae*	FBD
Myb_DNA-bind_3	*F. vesca*, *Z. mays*	*Poaceae*, *Rosaceae*	Myb/SANT-like DNA-binding domain
PA	*M. domestica*, *V. vinifera*	*Rosaceae*, *Vitaceae*	PA domain
PAH	*A. thaliana*, *Z. mays*	*Brassicaceae*, *Poaceae*	Paired amphipathic helix repeat
PARP	*A. lyrata*, *T. urartu*	*Brassicaceae*, *Poaceae*	Poly(ADP-ribose) polymerase catalytic domain
PPR_1	*B. napus*, *M. domestica*	*Brassicaceae*, *Rosaceae*	PPR repeat
PTEN_C2	*E. grandis*, *T. urartu*	*Myrtaceae*, *Poaceae*	C2 domain of PTEN tumour suppressor protein
Proteasome_A_N	*M. domestica*, *P. trichocarpa*	*Rosaceae*, *Salicaceae*	Proteasome subunit A N-terminal signature
RVT_2	*G. max*, *T. cacao*	*Fabaceae*, *Malvaceae*	Reverse transcriptase (RNA-dependent DNA polymerase)
S_locus_glycop	*G. max*, *V. vinifera*	*Fabaceae*, *Vitaceae*	S-locus glycoprotein family
Sugar_tr	*B. rapa*, *M. domestica*	*Brassicaceae*, *Rosaceae*	Sugar (and other) transporter
TPR_11	*P. patens*, *T. cacao*	*Funariaceae*, *Malvaceae*	TPR repeat
TPR_12	*C. subellipsoidea*, *V. carteri*	*Coccomyxaceae*, *Volvocaceae*	Tetratricopeptide repeat
UPF0114	*L. usitatissimum*, *M. truncatula*	*Fabaceae*, *Linaceae*	Uncharacterized protein family, UPF0114
XH	*B. rapa*, *T. urartu*	*Brassicaceae*, *Poaceae*	XH domain
zf-CCHC_4	*F. vesca*, *T. urartu*	*Poaceae*, *Rosaceae*	Zinc knuckle
zf-RING_2	*F. vesca*, *T. aestivum*	*Poaceae*, *Rosaceae*	Ring finger domain

^a^Integrated domains present across at least two plant families. Additional file 3 contains the full list of integrated domains. Additional file 6 contains list of domains for each protein

We have used publicly available RNA-seq data to further test which of the predicted fusions are supported by the expression evidence in two newly sequenced crop species, *B. rapa* and bread wheat, *T. aestivum*. Manual examination of RNA-seq alignments showed that in *B. rapa* 20 out of 25 genes were expressed and only 8 genes (40 %) had reads spanning exons connecting the predicted NLR and its ID (Additional files 10 and 11). In *T. aestivum*, 25 out of 43 genes showed strong expression, and 20 out of 25 (80 %) of the expressed fusions were strongly supported by RNA-seq reads (Additional file 12). For wheat (*T. aestivum* and *T. urartu*), we have confirmed four NLR-IDs by amplification from cDNA and sub-cloning (Additional file 13). As these are examples of the draft genome sequences, our manual analyses confirm that many of the detected fusions are real and not due to miss-assembly or annotation errors, although more experimental evidence is needed to test all predictions.

We used Fisher’s exact test to see if the detected protein domains are overrepresented in NLR-IDs compared to the rest of the genomes (Additional file 14). We observed that indeed most of the domains have a significant association with the NLR-ID set (*P* value <0.05). However, the integration event by itself does not signify functional relevance. Therefore, we tested which of the fused domains are found throughout several plant families, which could indicate either recurrent integration or retention of ancient fusions.

### Re-occurring and ancient domain integrations

**Fig. 2:**
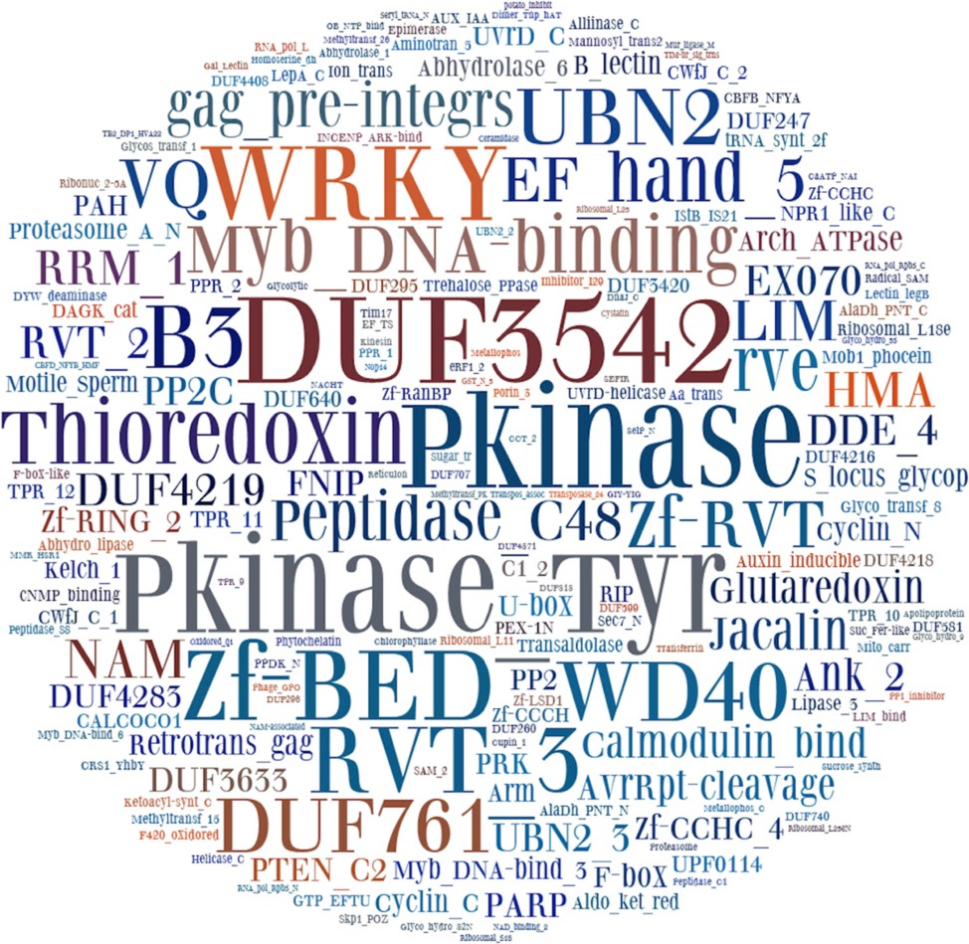
Word cloud analysis of the putative sensor domains found in fusion to NLRs. The word cloud represents relative abundance of different domains found in fusion. To correct for biases of sequencing particular plant families, word cloud was constructed on the plant family level (list of all domains occurring in NLR-ID in each plant family)

Overall, we found 265 distinct integrated domains in 750 NLR proteins. Comparing NLR-IDs across species, we observed that 61 distinct Pfam domains are present in plants belonging to at least two different families. These prevalent domains are enriched in protein activities associated with protein kinases, DNA-binding domains and protein-protein interactions (Fig. 2, Table 1). Domains associated with retrotransposons are also found in fusion with NLRs ubiquitously across plants (Fig. 2, Table 1). Retrotransposons have been shown to have a role in R-gene diversity and function [57], yet currently we do not have enough evidence to suggest transposon activity plays a role in generating NLR-IDs.

While some domains showed clear recurrent integration (i.e. WRKYs, see detailed analyses in a later section), a few proteins suggested ancient integration events. For example, an NLR-jacalin fusion is present in 6 out of 8 grasses and we confirmed this fusion by sub-cloning from cDNA of *T. aestivum*. As the grasses (*Poaceae*) separated from a common ancestor 70–55 million years ago [58], the NLR-jacalin is likely to be an ancient fusion event. Another validated fusion, NLR-Exo70 is present in two analyzed wheat species as well as barley, but functions as separate proteins in rice. Therefore, the NLR-Exo70 fusion event likely occurred at the split between *Triticeae* and *Oryza*, 40 million years ago.

Together, the results show that NLR-IDs are present in the genomes of most flowering plants, and we could detect that at least 61 integrated domains were selected by more than one plant family. These data suggest that plants share a common mechanism of NLR evolution through gene fusions. We hypothesize that these newly integrated domains serve as baits for the pathogen and that the same pathways are targeted across multiple plant species.

### Integrated domains overlap with host targets of pathogen effectors

**Fig. 3:**
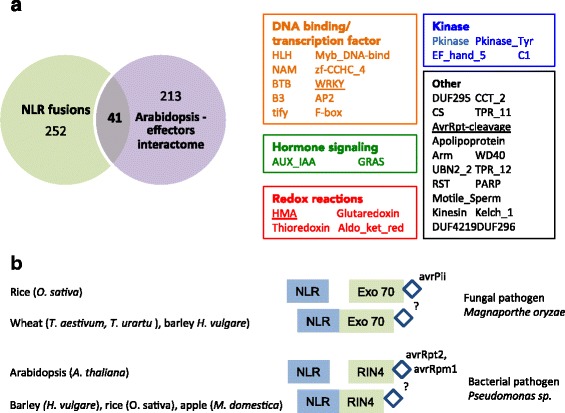
Overlap between IDs and domains present in host targets of plant pathogens. **a** An overlap between NLR-IDs from this study and functional domains present in the published Arabidopsis “effector interactome”. **b** Two examples of NLRs and their guardees, RIN4 and Exo70, that are known to be targeted by effectors in rice and Arabidopsis and which are found as fusions in other plant species

Several studies set out to reveal host targets of phytopathogen effectors by conducting genome-wide effector interactome screens, such as yeast two-hybrid screens against Arabidopsis proteins [47, 48]. We examined the overlap between protein domains fused to plant NLRs and protein domains found to interact with effectors. To ensure uniform analyses, we annotated domains of the predicted effector targets using our pipeline. We found that 41 out of 213 domains that are found in the Arabidopsis interactome studies are also present in NLR-IDs (Fig. 3a, Table 2). Overlapping domains include protein kinases, DNA-binding and transcription factor proteins, and proteins involved in redox reactions as well as hormone signaling and cytoskeleton (Fig. 3a, Table 2).

**Table 2:** Pathogenic effectors, their previously identified interacting Arabidopsis proteins and corresponding domains that were also detected in NLR-IDs

Effector	Interacting Arabidopsis gene	Common domain with NLR-ID	Domain description
OEC55	AT1G04690	Aldo_ket_red	Aldo/keto reductase family
ATR1_group	AT4G00980	DUF4219	Domain of unknown function (DUF4219)
ATR1_group	AT4G00980	UBN2_2	Gag-polypeptide of LTR copia-type
ATR1_group	AT4G00980	zf-CCHC_4	Zinc knuckle
ATR13_group	AT5G66560	BTB	BTB/POZ domain
ATR13_group	AT5G52750	HMA	Heavy metal-associated domain
avrB_group	AT3G25070	AvrRpt-cleavage	Cleavage site for pathogenic type III effector avirulence factor Avr
avrB_group	AT1G14920	GRAS	GRAS domain family
avrB_group	AT2G47060	Pkinase	Protein kinase domain
avrB_group	AT3G17410	Pkinase_Tyr	Protein tyrosine kinase
avrC_group	AT4G17680	Apolipoprotein	Apolipoprotein A1/A4/E domain
avrPto_group	AT5G22355	C1_2	C1 domain
avrPto_group	AT5G22355	C1_3	C1-like domain
avrPto_group	AT4G11890	Pkinase	Protein kinase domain
avrPto_group	AT3G48150	TPR_11	TPR repeat
AvrRps4_Pph_1448A	AT4G11070	WRKY	WRKY DNA-binding domain
AVRRPT2_group	AT4G00710	Pkinase_Tyr	Protein tyrosine kinase
AVRRPT2_group	AT4G00710	TPR_11	TPR repeat
HARXL10_WACO9	AT1G50420	GRAS	GRAS domain family
HARXL106_group	AT1G09270	Arm	Armadillo/beta-catenin-like repeat
HARXL106_group	AT4G02150	Arm	Armadillo/beta-catenin-like repeat
HARXL106_group	AT1G32230	PARP	Poly(ADP-ribose) polymerase catalytic domain
HARXL106_group	AT1G32230	RST	RCD1-SRO-TAF4 (RST) plant domain
HARXL14	AT5G66200	Arm	Armadillo/beta-catenin-like repeat
HARXL149	AT4G35580	NAM	No apical meristem (NAM) protein
HARXL16	AT1G18400	HLH	Helix-loop-helix DNA-binding domain
HARXL16	AT4G32570	tify	tify domain
HARXL21	AT1G15750	WD40	WD domain, G-beta repeat
HARXL44	AT4G25920	DUF295	Protein of unknown function (DUF295)
HARXL44	AT4G16380	HMA	Heavy metal-associated domain
HARXL45_group	AT4G02550	Myb_DNA-bind_3	Myb/SANT-like DNA-binding domain
HARXL68	AT1G45145	Thioredoxin	Thioredoxin
HARXL68	AT5G42980	Thioredoxin	Thioredoxin
HARXL73	AT4G39050	Kinesin	Kinesin motor domain
HARXL79	AT5G56950	NAP	Nucleosome assembly protein (NAP)
HARXLL445_group	AT2G35500	CS	CS domain
HARXLL470_WACO9	AT5G49000	F-box	F-box domain
HARXLL470_WACO9	AT5G49000	Kelch_1	Kelch motif
HARXLL470_WACO9	AT1G79430	Myb_DNA-binding	Myb-like DNA-binding domain
HARXLL492	AT3G60600	Motile_Sperm	Major sperm protein (MSP) domain
HARXLL60	AT2G23290	Myb_DNA-bind_6	Myb-like DNA-binding domain
HARXLL73_group	AT1G03960	EF_hand_5	EF-hand domain pair
HARXLL73_group	AT4G26110	NAP	Nucleosome assembly protein (NAP)
HARXLL73_group	AT5G56290	TPR_11	TPR repeat
HOPAB_group	AT3G57720	Pkinase	Protein kinase domain
HOPAB_group	AT3G46370	Pkinase_Tyr	Protein tyrosine kinase
HOPAB_group	AT3G27960	TPR_12	Tetratricopeptide repeat
HopBB1_Pmo_M301020	AT3G17860	CCT_2	Divergent CCT motif
HopBB1_Pmo_M301020	AT3G17860	tify	tify domain
HOPD1_group	AT5G22290	NAM	No apical meristem (NAM) protein
HOPF_group	AT2G04740	BTB	BTB/POZ domain
HOPH1_group	AT5G43700	AUX_IAA	AUX/IAA family
HopP1_Pto_DC3000	AT4G36540	HLH	Helix-loop-helix DNA-binding domain
HOPR1_group	AT5G60120	AP2	AP2 domain
HopX_group	AT5G13810	Glutaredoxin	Glutaredoxin
OEC115	AT4G28640	AUX_IAA	AUX/IAA family
OEC115	AT5G08130	HLH	Helix-loop-helix DNA-binding domain
OEC115	AT3G21490	HMA	Heavy metal-associated domain
OEC115	AT3G10480	NAM	No apical meristem (NAM) protein
OEC45	AT1G63480	DUF296	Domain of unknown function (DUF296)
OEC45	AT4G00120	HLH	Helix-loop-helix DNA-binding domain
OEC45	AT1G12520	HMA	Heavy metal-associated domain
OEC45	AT3G22420	Pkinase	Protein kinase domain
OEC59	AT4G08320	TPR_11	TPR repeat
OEC67	AT1G25550	Myb_DNA-binding	Myb-like DNA-binding domain
OEC78	AT4G30080	AUX_IAA	AUX/IAA family
OEC78	AT4G30080	B3	B3 DNA-binding domain
OEC78	AT4G02590	HLH	Helix-loop-helix DNA-binding domain

A random protein set sampled from all plant proteomes could have domains in common with the Arabidopsis interactome. Some domains, such as protein kinases and Myb family DNA-binding domains, are indeed prevalent in plant genomes, and using 5 % confidence intervals, we cannot exclude a possibility of a random overlap. However, for the majority of domains, we find a significant overlap between effector targets and domains in fusions (*P* <0.05) (Additional file 14). Overall, this strong overlap indicates that protein domains fused to NLRs could be effector targets. Conceivably, effector targets not detected in our survey could occur as fusions in as yet uncharacterized plant species or sub-species. Future effector interactome screens are needed to test the identified NLR-IDs.

Overlap of IDs with effector targets is further exemplified by presence of well-characterized guardees on our fusions list. A recently found interaction between rice blast (*M. oryzae*) effector AvrPii and rice exocyst complex factor Exo70 is in line with our finding of an NLR-Exo70 fusion in wheat (Fig. 3b, Table 1). Wheat blast also caused by variants of the *M. oryzae* species might be harboring an effector recognized by this fusion. Alternatively, NLR-Exo70 in wheat might be the basis for host specificity of the rice blast pathogen. One of the most studied effector targets, RIN4, which interacts with several NLRs, including RPS2 and RPM1 in a classic guard/guardee system, is found as an NLR-RIN4 fusion in several species, including barley, rice and apple (Fig. 3b, Tables 1 and 2). These findings further support the links between guardees and integrated sensor domain models, in which a fusion reveals a previously interacting NLR and guardee that are now also linked together genetically.

### NLR-integrated kinase domains are frequent and intact

**Fig. 4:**
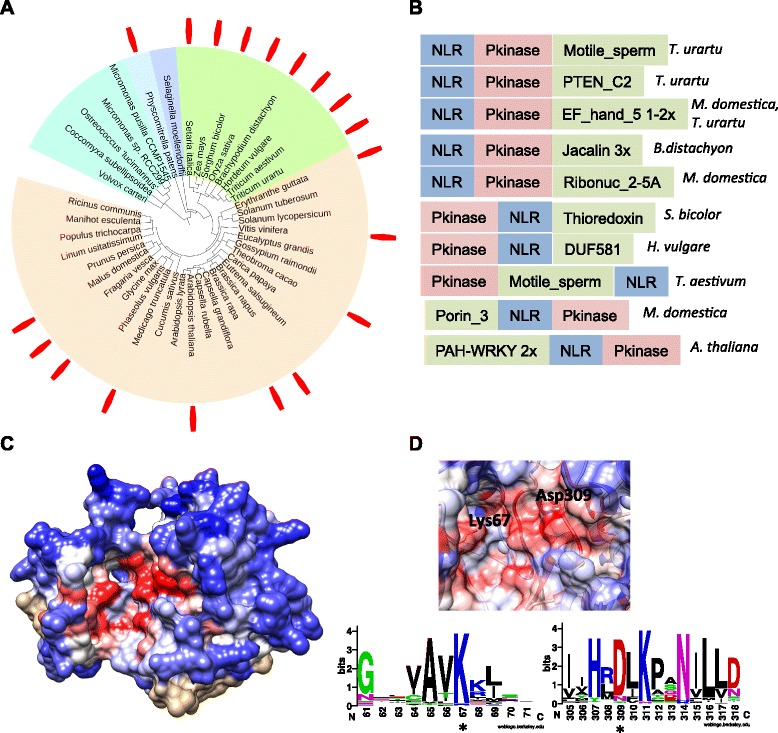
Domain architectures and structural analyses of NLR-kinase fusions. **a** Distribution of identified integrated kinase domains. **b** Complex fusions of domains on top of NLR-kinases. **c** Three-dimensional structural model of the kinase domain of an Arabidopsis NLR-kinase At4G12020 (aa 8–258) modelled after the best structural match, human serine/threonine protein kinase PAK 6 (PDB: 2C30). Conservation profile across all plant kinases found in NLR-IDs is overlapped on the structure, with most conserved residues depicted in red and most divergent in blue. **d** Zoom-in on the active site and its critical lysine and aspartate residues and a corresponding alignment logo show that the active site of kinases is completely conserved across all fusions

The most abundant class of NLR-fusion is the protein kinase domain found as early as in mosses and in 161 NLR proteins across 19 species and 8 plant families (Fig. 4a, Table 1). Both serine and tyrosine kinases are present, either as amino-terminal or carboxyl-terminal fusions (Additional files 6 and 8). A class of kinases called non-RD kinases are known to function in the immune pathways in both plants and mammals and are also often found in the receptor-like kinases that transduce PAMP-triggered immunity [59]. We examined the kinase motifs in NLR-IDs and observed that both RD and non-RD kinases are present.

Interestingly, a protein kinase was associated with another domain fusion in 14 different combinations (Fig. 4b). Some domain combinations are known modifiers of protein kinase activity; for example, the kinase + EF_hand is diagnostic of a Ca^2+^-dependent protein kinase that was part of a single gene before fusion with NLR. Other combinations likely represent sequential fusion events, such as a kinase-NLR-NPR1 fusion in *T. urartu* or an NLR-kinase-WRKY fusion in *A. thaliana* (Fig. 4b). There could be two explanations for such complex fusions. The kinase domains in the fusions would act as “sensors” for the effectors and double fusions would be simple stacks of different sensor domains. Alternatively, the kinases represent a class of signaling domains recruited by NLRs and the additional domains are operative enzymes that function as “integrated” sensors. Given the examples of PBS1 and Pto, two protein kinases that are guardees, it is most likely that the former hypothesis is true and that at least some of the kinase fusions are integrated sensors for the effectors.

**Fig. 5:**
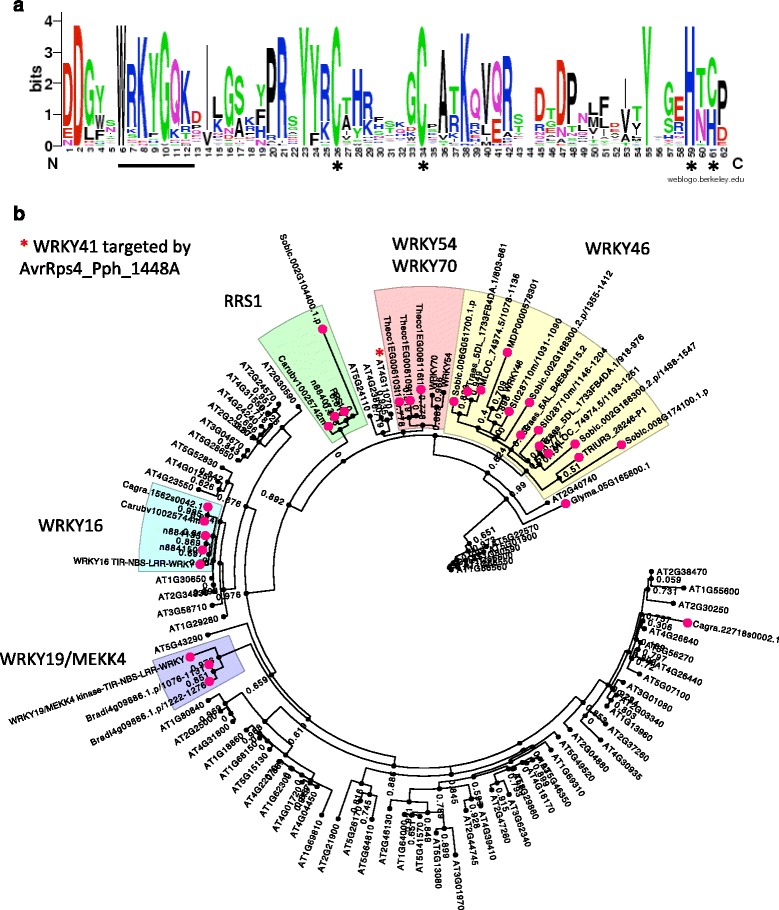
Evolutionary analyses of the WRKY domain in NLR-WRKYs family. **a** Multiple sequence alignment logo of the WRKY domains found in all NLR-IDs shows conserved core structural tryptophan and incomplete conservation of tyrosine and lysine that have been shown to be essential for recognizing the W-box DNA. **b** Maximum likelihood phylogeny of all Arabidopsis WRKY domain-containing proteins (black nodes) and the WRKYs detected as fusions in all flowering plants (strawberry nodes). Distinct Arabidopsis WRKY clades that form fusions are highlighted as the following: green, RRS1 clade; yellow, WRKY46; pink, WRKY70 and WRKY54; blue, WRKY16; and purple, WRKY19/MEKK4. Arabidopsis WRKY41 known to be the host target is marked with red asterisk

The current integrated decoy model suggests that the fused proteins might lose their biochemical activity after integration while retaining effector-binding properties [25]. To test whether NLR-kinase fusions follow the current model of integrated decoy, we have tested whether the kinase activity is likely to be conserved. After aligning all kinase regions from NLR-IDs, we examined conservation of active site region and catalytic residues. We explored sequence conservation by mapping alignment of all kinases found in NLRs on the 3D structural model of the kinase, with the active site conserved (red) while most of the other regions are variable (blue) (Fig. 5b). The catalytic lysine and aspartate are also conserved in all kinases as can be seen from the structure as well as alignment consensus logo (Fig. 5c). Overall, these data indicate that the kinases fused with NLRs encode intact full-length kinase domains that are potentially catalytically active.

### WRKY transcription factor integration into NLRs occurred independently in several lineages of plants

The WRKY family of transcription factors is large and its members can be positive or negative regulators of both PTI and ETI [3], or in other plant signaling networks. In *Arabidopsis*, more than 70 % of WRKY genes are responsive to pathogen infection and salicylic acid treatment [60, 61], suggesting a major role of these proteins in plant defense. We have found the WRKY domain to be present in 35 NLR-ID genes from 13 plant species, in monocots and dicots, including previously reported *A. thaliana*, *A. lyrata*, *Fragaria vesca*, *Capsella rubella*, *Glycine max*, *Theobroma cacao*, *Sorghum bicolor*, *Setaria italica*, *O. sativa* [62] as well as in *M. domestica*, *Conradina grandiflora*, *B. distachyon*, *Hordeum vulgare*, *T. aestivum* and *T. urartu* (Table 1, Additional file 15). Similar to Rinerson et al. [62], we also detected an NLR-WRKY fusion in *Panicum virgatum*, but did not include it in our high-throughput analyses due to current restrictions on using genome-wide data for this species. The only reported NLR-WRKY that was not found in our screen is GrWRKY1 from *Gossypium raimondii*, which is according to the authors of the study “truncated and difficult to classify” [62].

Our protein sequence alignment of 7 domain regions from NLR-IDs showed that all sequences contain functional Zn^2+^-binding motifs CX_4-5_CX_22-23_HXH or CX_7_CX_23_HXC (Fig. 5a). While the protein core stabilizing tryptophan is conserved, the DNA-binding motif of WRKYG[Q/K]K is mutated in several fusion proteins (Fig. 5a), including variants of the tyrosine and lysine that have been shown to be essential for recognizing the W-box DNA element [63]. The group I WRKY NLR-fusion proteins, which contain 2 × WRKY motifs, often show mutations in the second critical motif. Given this evidence, we cannot exclude that in several NLR-IDs, WRKY region is indeed a “decoy” protein deficient in its DNA-binding activity.

Our data also support that the NLR-WRKY fusions occurred independently in several lineages, including both monocots and dicots. The phylogenetic analyses of all fused WRKY domains together with all WRKY proteins from Arabidopsis showed that fusions arose at least five times and involved homologs of RRS1, WRKY16, WRKY19, WRKY46 and WRKY54/70 (Fig. 5b, Additional file 16). Interestingly, the WRKY46 fusion appears to be specific to monocots, but it is widespread and potentially an old fusion event as it is present in wheat (*T. aestivum* and *T. urartu*), barley (*H. vulgare*), sorghum (*S. bicolor*) and *S. italica*. It has been reported that WRKY46 plays a role in basal resistance against bacterial pathogens and is specifically induced by salicylic acid [60, 64, 65], and is therefore a plausible pathogen target. The WRKY54/70 cluster together with the NLR-WRKY fusions in *T. cacao*, and in Arabidopsis they have been implicated in resistance as a WRKY54/70 double mutant shows increased susceptibility to Pseudomonas infection [66]. Next to the WRKY54/70 is the WRKY41 (Fig. 5b), which is targeted by a number of bacterial effectors in the Arabidopsis interactome yeast two-hybrid screen (Table 2). Finally, WRKY19 (also known as MEKK4) represents a complex WRKY-NLR-kinase fusion and the clustering with similar NLR-IDs in *Brachypodium* points at a common “fusion” of immunity genes across both dicots and monocots.

This example of WRKY transcription factor family fusions across plants exemplifies recurrent fusions of the same protein family members across different lineages. It is clear that some of the fusions are more commonly found in monocots (i.e. WRKY46) while others are spread across phyla and point to the common convergent targets of pathogens infecting diverse evolutionary hosts. While most WRKYs in fusions have all the signatures of the functional WRKY transcription factors, gradual loss of activity in the “decoys” cannot be rejected as some of the integrated WRKY proteins show loss of the conserved critical residues.

## Conclusions

Interaction of the effectors with fusion domains in NB-LRRs for both Arabidopsis RPS4/RRS1 and rice Pik-1, RGA4/RGA5, represented the first evidence for the “integrated decoy/sensor” pathogen recognition model, whereby the atypical domain acts as bait/trap for effector perception. Our findings of other protein domains fused to NB-LRR proteins in various plant genomes provide a new perspective on effector targets and the nature of pathogenicity. As we found NLR-IDs in most plant species, we can predict that pathogen recognition through “integrated decoy/sensor” receptors is an evolutionarily conserved mechanism of NLR diversification in flowering plants.

**Fig. 6:**
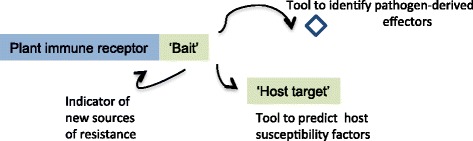
Summary of the information encoded in the discovered NLRs that possess “integrated domains”

Overlap between fusions and effector targets point to the multiple levels of information encoded in NLR-IDs (Fig. 6). Presented NLR-IDs are likely to be molecular sensors of the effectors, so they can also be exploited to identify and validate pathogen-derived virulence factors. For many pathogens, researchers have now accumulated long lists of predicted effector molecules that are likely to be secreted or translocated inside plant cells. Systematic analyses of these effectors against the NLR-IDs in either proteomic or yeast two-hybrid assays would allow for prioritization and validation of pathogen effectors. These validation tools represent an important milestone for deciphering pathogen arsenals and identifying new sources of disease resistance.

Extrapolating from the known mechanistic analyses, we predict that the NLR-IDs reveal not only disease resistance genes that use baits for catching the pathogen, but also potentially previously unknown effector targets inside the host. Therefore, investigation of identified fusions and tracing their origin will significantly contribute to the identification of host “susceptibility” genes.

In the future, it would be important to continue examining NLR-IDs both across plants and within each plant family to enrich our knowledge of the evolutionary history of NLR proteins. We need to understand the mechanisms leading to fusion events, and how often fusions occur in different plant lineages and across NLR families. It appears that polyploidization and ancient polyploidization played a major role in expanding the number of NLRs and consequently the number of NLR-IDs. It would be important to test if there are any genetic or molecular signatures that enable NLR platforms to be more prone to tolerating new fusions. This information will give us a better understanding of how plant immune receptors evolve to withstand pathogen pressure and can lead to new ways of engineering disease resistance.

## Methods

### Phylogeny of plant species

Phylogeny of all plant species was constructed using PhyloT program (http://phylot.biobyte.de/), using NCBI taxonomy identification numbers for each species and visualized with iTOL program. Polyploidization and ancient polyploidization events were inferred from Jiao et al. [50] and Adams et al. [51] as well as the CoGe database (https://genomevolution.org/wiki/index.php/Plant_paleopolyploidy).

### Domain annotations and high-throughput identification of gene fusions

Domain annotations in all species were performed on the currently available proteome predictions, which included Phytozome v10 genomes [67] available for analyses without restrictions as well as recently published wheat, barley and brassica datasets (Additional file 1). Proteins from each species were passed through uniform Pfam [68] domain identification pipeline based on the run_pfam.pl script distributed together with *PfamScan* software (HMMER2.0 package [69], e-value cutoff 1e-3). Resulting annotations were parsed using K-parse_Pfam_domains_v3.1.pl script generated in this study and available from GitHub (https://github.com/krasileva/plant_rgenes). Only highest scoring non-overlapping domains were retained for each protein. Proteins containing NB-ARC domains were extracted and checked for additional fused domains with K-parse_Pfam_domains_NLR-fusions-v2.2.pl (https://github.com/krasileva/plant_rgenes).

After filtering out classic NLR domains, such as TIR (PF01582), TIR2 (PF13676), LRR (CL0022) and RPW8 (PF05659), all other domains were considered for further analyses and a summary table of domains found in each plant species and each plant family was generated. To test for significance of overrepresentation of each domain in the fusion set, we applied the hypergeometric Fisher’s exact test as implemented in K-parse_Pfam_domains_NLR-fusions-v1.0.pl (https://github.com/krasileva/plant_rgenes). Fusions in four distinct plant clades, including brassica, tomato, wheat and soybean, were manually curated using manual selection and screening of all the annotated, predicted and not predicted NB-LRRs from each species using the HMMER, SMART and BLASTP online programs (Additional file 8) showing less than 10 % of false positives in our high-throughput analyses.

In order to determine the expression of and provide an evidence for the predicted NLR-IDs, we obtained RNA-seq reads derived from 9-day-old seedlings of *B. rapa* cv. Chiifu (DRX012760/BioSample: SAMD00003761) as well as RNA-seq from leaf samples from *T. aestivum* cv. Chinese Spring (sample: ERS399938). For *B. rapa*, the reads were then aligned back to NLR-fusion genes using TOPHAT 2.1.0 [70]. For *T. aestivum* analyses, the reads were aligned back to the full genome [53] using TOPHAT 2.1.0 [70]. All alignments were performed with -r 300 --mate-std-dev = 20; the rest of the parameters at default values. The alignments in BAM format were then used to visualize with the Integrated Genomics Viewer (IGV) tool [71] or Tablet [72]. We then manually analyzed the splice junctions and their correspondence with the predicted gene structures as well as reads spanning exons coding for predicted protein domains, particularly the fusions.

### Word cloud

Prevalence of domain fusions across plant families (each domain counted only once per family) was visualized as a word cloud at http://www.tagxedo.com/ with the following non-default parameters that preserve exact names of all domains: punctuation, yes; numbers, yes; remove common words, no; and combine related words, no.

### Calculating overlap with interactome datasets

Amino acid sequences of the proteins reported as effector interactors [47] were annotated using the same Pfam annotation pipeline as above. The overlap of domains co-occurring in the interactors and protein fusions were manually examined. The statistical significance of the enrichment of the domains was tested using the hypergeometric Fisher’s exact test, which tested for significance of overrepresentation of each domain in the fusion set and implemented in K-parse_Pfam_domains_NLR-fusions-v1.0.pl (https://github.com/krasileva/plant_rgenes).

### Protein family sequence alignment, structural modeling and phylogenetic analyses

For each protein family of interest, the amino acid sequences of all fusion-containing proteins were extracted using K-get_fasta_from_ids.pl and aligned together on the corresponding Pfam HMM profile using the *hmmalign* program (HMMER2.0) [69]. The alignment was converted from Stockholm to FASTA format using bioscripts.convert tools v0.4 (https://pypi.python.org/pypi/bioscripts.convert/0.4). The alignment was examined with Belvu program and trimmed to the domain borders. Trimmed sequences were then re-aligned with MUSCLE [73].

The evolution of TIR_2 domains was inferred with MEGA5 [74] using the maximum likelihood method based on the Poisson correction model [75]. The bootstrap consensus tree was inferred from 400 bootstrap replicates [76]. Initial tree(s) for the heuristic search were obtained automatically as follows: when the number of common sites was <100 or less than one-fourth of the total number of sites, the maximum parsimony method was used; otherwise BIONJ method with MCL distance matrix was used. The tree was drawn to scale, with branch lengths measured in the number of substitutions per site. The analysis involved 74 amino acid sequences. All positions were evaluated regardless of alignment gaps, missing data and ambiguous bases. There were a total of 75 positions in the final dataset.

Structural modelling of the kinase domain was performed with Phyre2 using amino acid sequence of the kinase domain from At4G12020 (aa 8–258) and the best structure (highest percent identity, most sequence coverage) modelled after human serine/threonine protein kinase PAK 6 (PDB: 2C30) was picked as a template. The structure was visualized in Chimera [77] and amino acid conservation from multiple sequence alignment of all kinase fusions was mapped to the structure using “render by conservation” function with 0.017 and 0.85 conservation cutoffs. The alignment logo of the kinase active site was constructed with WebLogo (weblogo.berkeley.edu/logo.cgi). The phylogeny of WRKY transcription factors was constructed with PhyML method using Phylogeny.fr with SH-like approximate likelihood ratio test. The tree was annotated and visualized using FigTree v1.4.2 (http://tree.bio.ed.ac.uk/software/figtree/). WRKY alignment conservation logo plot was constructed with WebLogo.

## Availability of supporting data

The plant proteome datasets analyzed in this study were obtained from publicly available databases Phytozome v10 and Ensembl Plants, and are listed in Additional file 1. Specific sequences of NLR and NLR-ID proteins and corresponding domain architectures are available in Additional files 2, 3, 4, 5 and 7. All scripts written for this study are available from GitHub at https://github.com/krasileva/plant_rgenes. All additional files are supplied in standard formats (Excel, PDF and FASTA [in Unix line break format]). In the event that any additional file is not compatible for a user computer’s platform, please contact corresponding author: Ksenia.Krasileva@tgac.ac.uk.

## Additional files

Additional file 1:
**Plant datasets used in this study.** A table with the description of species, genome builds and databases from where they were retrieved. (XLSX 32 kb) Additional file 2:
**NLR and NLR-ID architectures detected in each species.** (XLSX 44 kb) Additional file 3:
**Summary of the integrated domains and their prevalence.** (XLSX 58 kb) Additional file 4:
**Protein ids and corresponding domains of putative NLRs from all plant species.** A table of domain architectures detected in all NB-ARC-containing proteins. (XLSX 953 kb) Additional file 5:
**Amino acid FASTA sequences of all putative NLRs.** Unix-encoded plain text file with FASTA sequences for all NB-ARC-containing proteins from Additional file 4. (TXT 14163 kb) Additional file 6:
**Protein ids and corresponding domains of putative NLR-IDs from all plant species.** Table of domain architectures detected in each protein classified as NLR-fusion. (XLSX 114 kb) Additional file 7:
**Amino acid FASTA sequences of putative NLR-IDs.** Unix-encoded plain text file with FASTA sequences for all NLR-fusion proteins from Additional file 6. (TXT 917 kb) Additional file 8:
**Manual verification of domains predicted by high-throughput scripts with webservers for brassica, tomato, wheat and soybean.** (XLSX 22 kb) Additional file 9:
**Phylogeny of TIR2 proteins from plants and phytopathogenic bacteria.** (PDF 447 kb) Additional file 10:
***B. rapa*** **fusion validation by RNA-seq.** Summary of manual validation of *B. rapa* NLR-IDs using RNA-seq data. (XLSX 36 kb) Additional file 11:
**Visual examples of validated** ***B. rapa*** **fusions.** (PDF 375 kb) Additional file 12:
***T. aestivum*** **fusion validation by RNA-seq.** Summary of manual validation of *T. aestivum* NLR-IDs using RNA-seq data. (XLSX 60 kb) Additional file 13:
**Cloned** ***T. aestivum*** **and** ***T. urartu*** **fusions.** Summary of *T. aestivum* and *T. urartu* fusions confirmed by sub-cloning from cDNA. (XLSX 23 kb) Additional file 14:
**Enrichment analyses of NLR-IDs.** Summary of hypergeometric tests for each domain present in NLR-IDs in each plant species. (XLSX 84 kb) Additional file 15:
**Visual representation of distribution of WRKY fusions across flowering plants.** (PDF 1729 kb) Additional file 16:
**Maximum likelihood phylogenetic tree of WRKY transcription factors in fusion with NLRs in all plants together with all other Arabidopsis WRKY proteins.** (TXT 4 kb)

## Abbreviations

CC: coiled coil
ETI: effector-triggered immunity
HMA: heavy metal-associated
ID: integrated domain
LRR: leucine-rich repeats
NB: nucleotide-binding
NCBI: National Center for Biotechnology Information
NLR: nucleotide-binding leucine-rich repeat
PAMP: pathogen-associated microbial pattern
PTI: PAMP-triggered immunity
TIR: Toll/interleukin-1 receptor/resistance protein
