# Supplementary figures and images for: Comparative analysis of plant immune receptor architectures uncovers host proteins likely targeted by pathogens
Source: BMC Biol. 2016 Feb 19;14:8. doi: 10.1186/s12915-016-0228-7 (PMC4759884; doi:10.1186/s12915-016-0228-7)

Bacterial TIR\_2

Plant TIR\_2

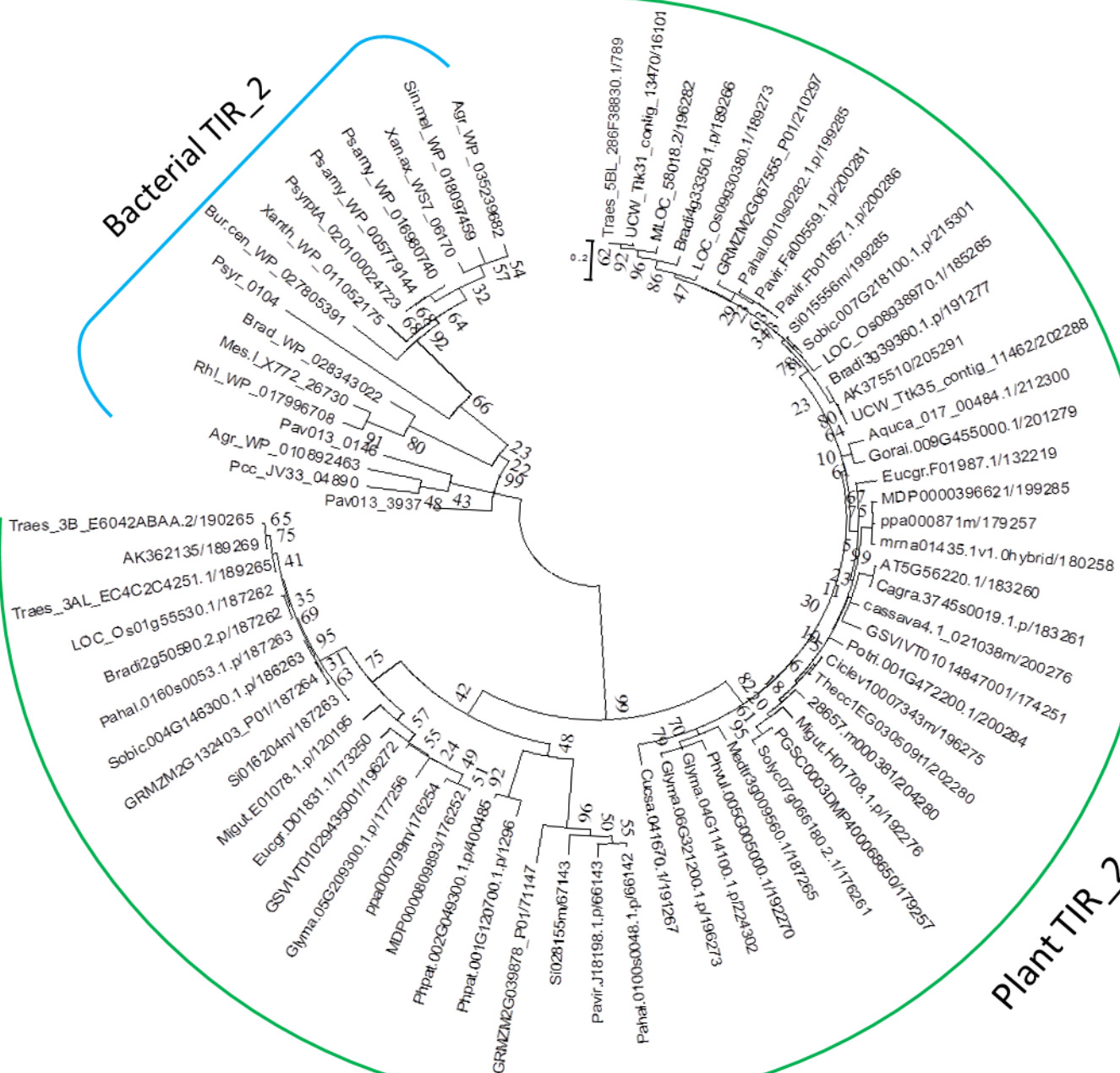

Supplement: Additional file 9: — Phylogeny of TIR2 proteins from plants and phytopathogenic bacteria. (PDF 447 kb) [file 12915_2016_228_MOESM9_ESM.pdf]

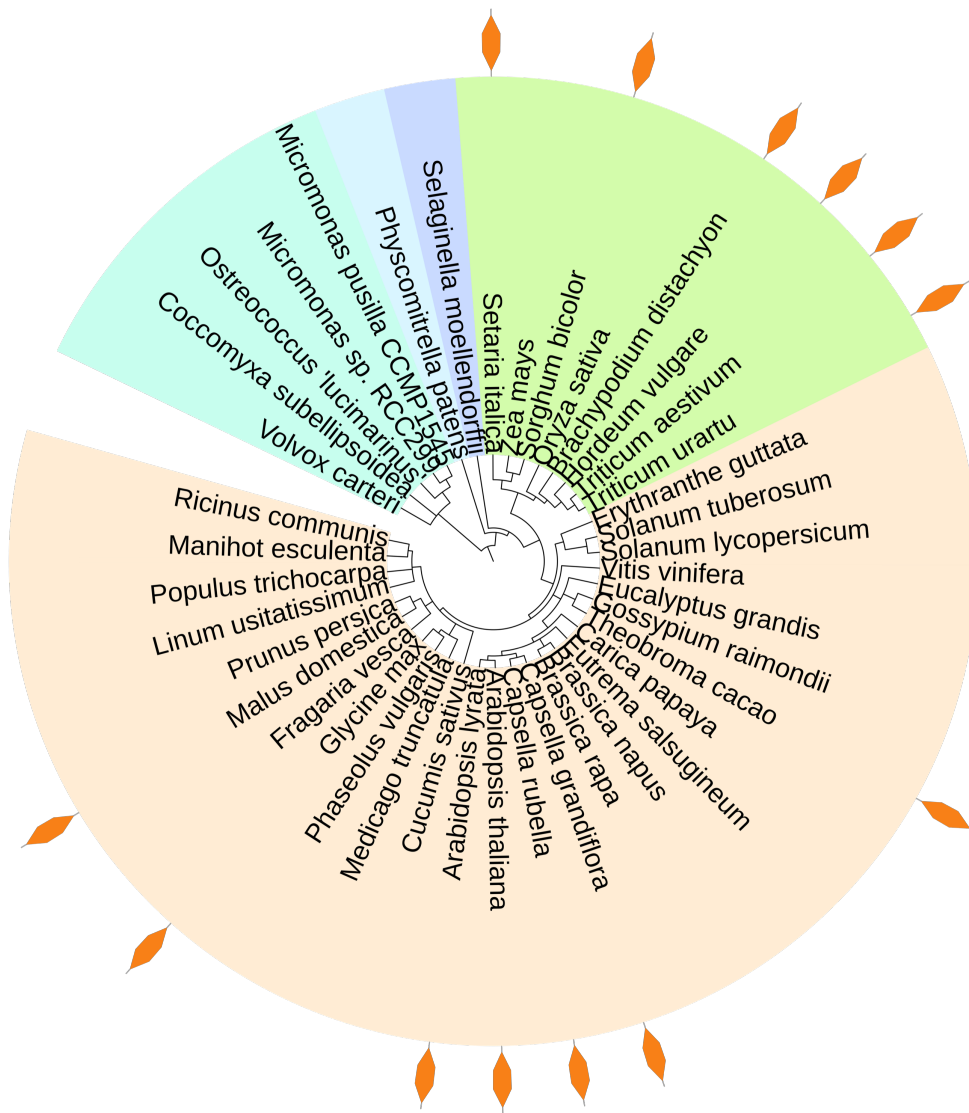

Supplement: Additional file 15: — Visual representation of distribution of WRKY fusions across flowering plants. (PDF 1729 kb) [file 12915_2016_228_MOESM15_ESM.pdf]
