# Supplementary material for: Comparative analysis of plant immune receptor architectures uncovers host proteins likely targeted by pathogens
Source: BMC Biol. 2016 Feb 19;14:8. doi: 10.1186/s12915-016-0228-7 (PMC4759884; doi:10.1186/s12915-016-0228-7)
Supplement: Additional file 11: — Visual examples of validated B. rapa fusions. (PDF 375 kb) [file 12915_2016_228_MOESM11_ESM.pdf]

Bra000758 is a part of a R-gene pair

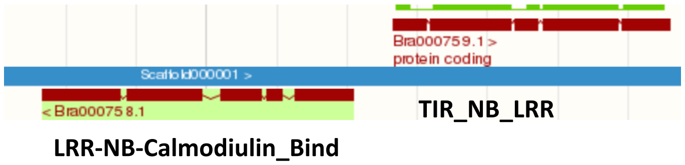

Bra000758

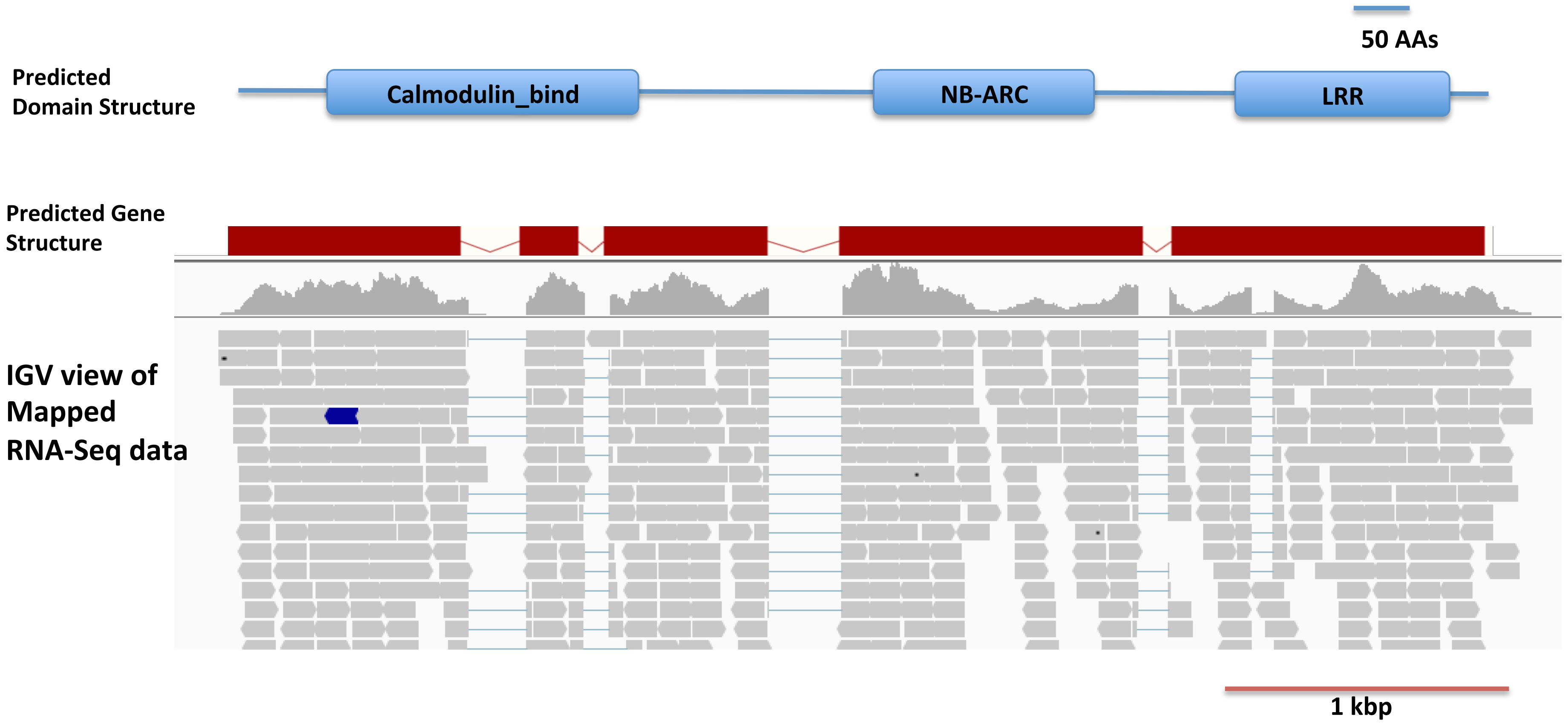

Bra003867

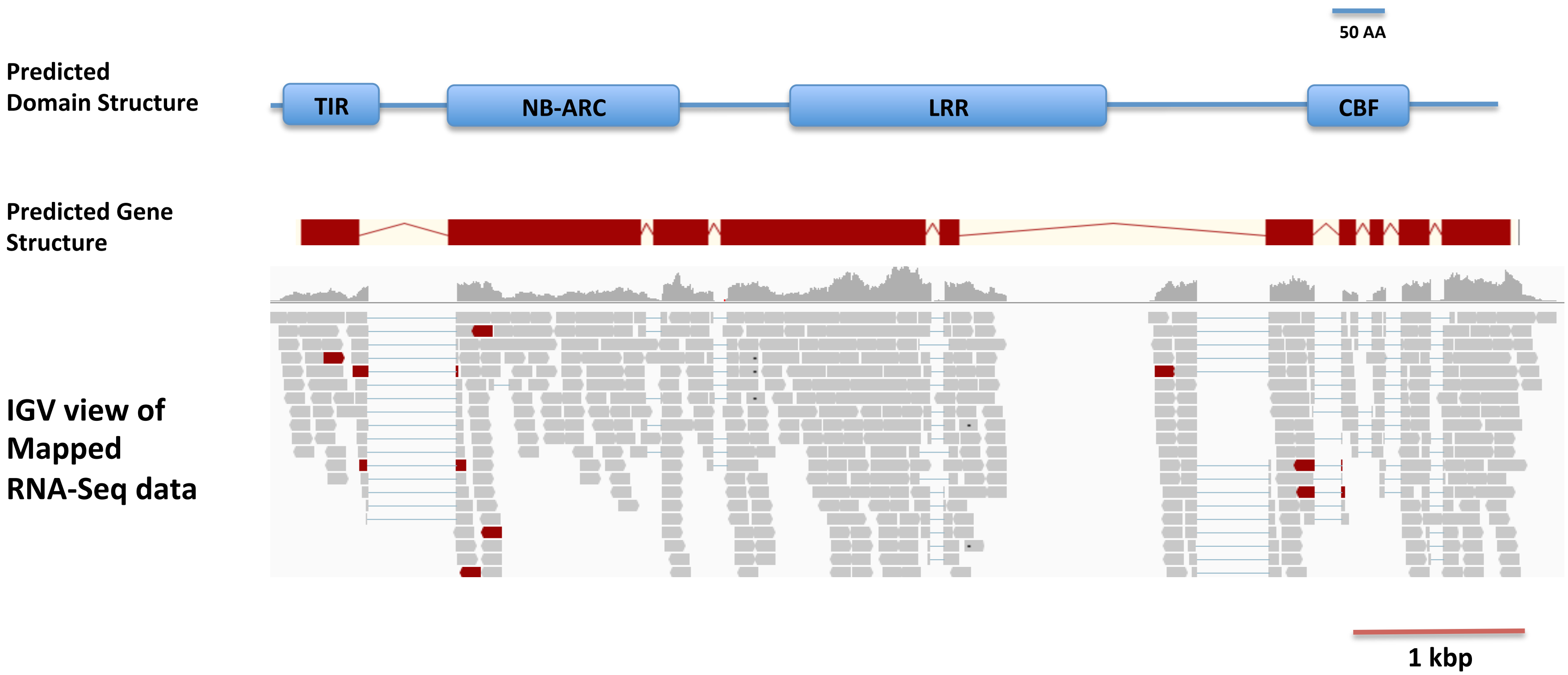

50 AA
Predicted Domain Structure
TIR
NB-ARC
LRR
CBF
Predicted Gene Structure
IGV view of Mapped RNA-Seq data
1 kbp
